# Supplementary material for: Lamprey Wound Healing and Regenerative Effects: The Collaborative Efforts of Diverse Drivers
Source: Int J Mol Sci. 2023 Feb 6;24(4):3213. doi: 10.3390/ijms24043213 (PMC9965152; doi:10.3390/ijms24043213)
Supplement: Supplementary file 1 [file ijms-24-03213-s001.zip › Supplementary Figure.pdf]

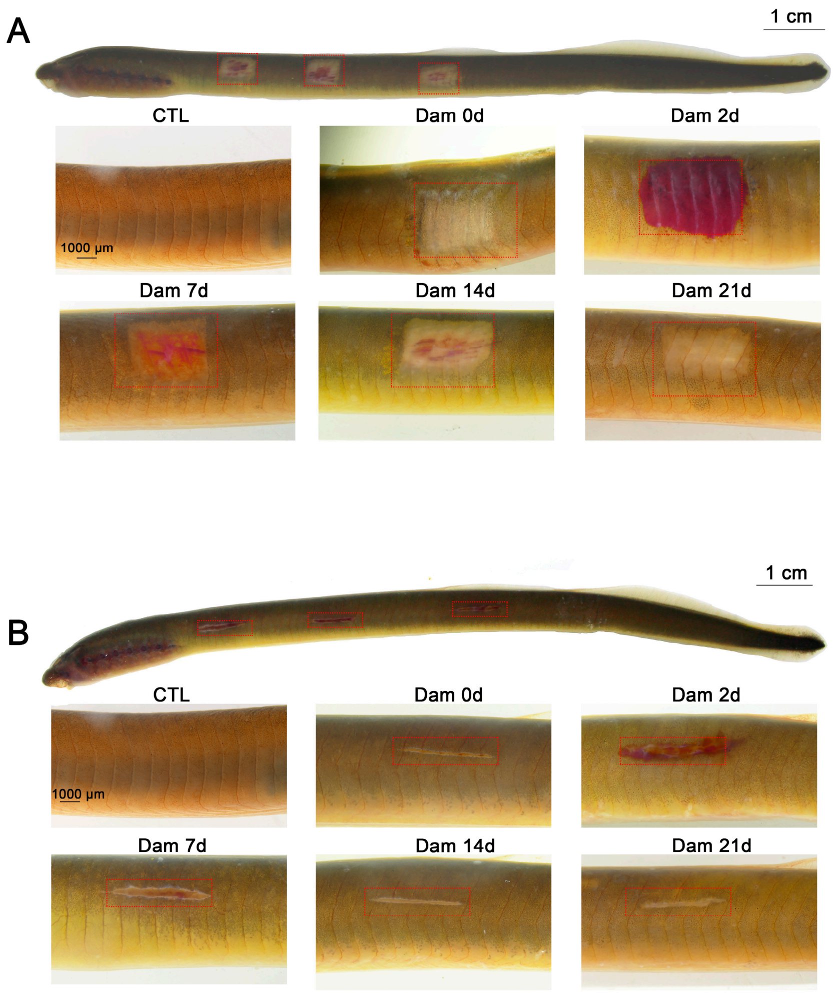

Supplementary Figure S1. Process of wound healing in lamprey. (A) Photograph of epidermal damage in larvae. From undamaged skin to 21 d after damage, hyperemia was more prominent; scale bars, 1000 μm. (B) Photograph of full-thickness skin damage to lamprey larva, from undamaged skin to 21 d after damage, with repair delays; scale bars, 1000 μ.

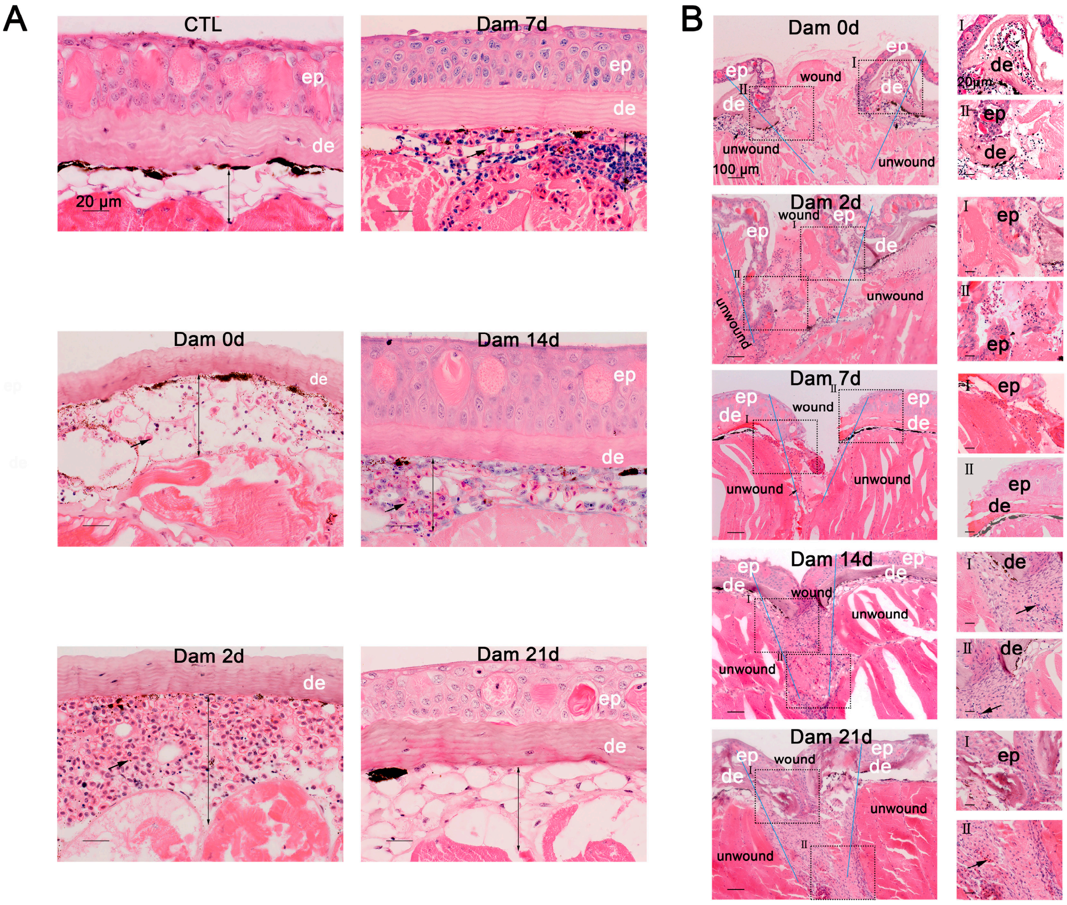

Supplementary Figure S2. H&E staining at all stages of skin damage. (A) Representative images of the skin healing process of larval lamprey after epidermal damage and RBC infiltration (black arrow). (B) Representative images of the skin healing process of larval lamprey after full-thickness skin damage. Deepening of the damage causes the repair process to be delayed. The right panel is a magnified view of the left panel.

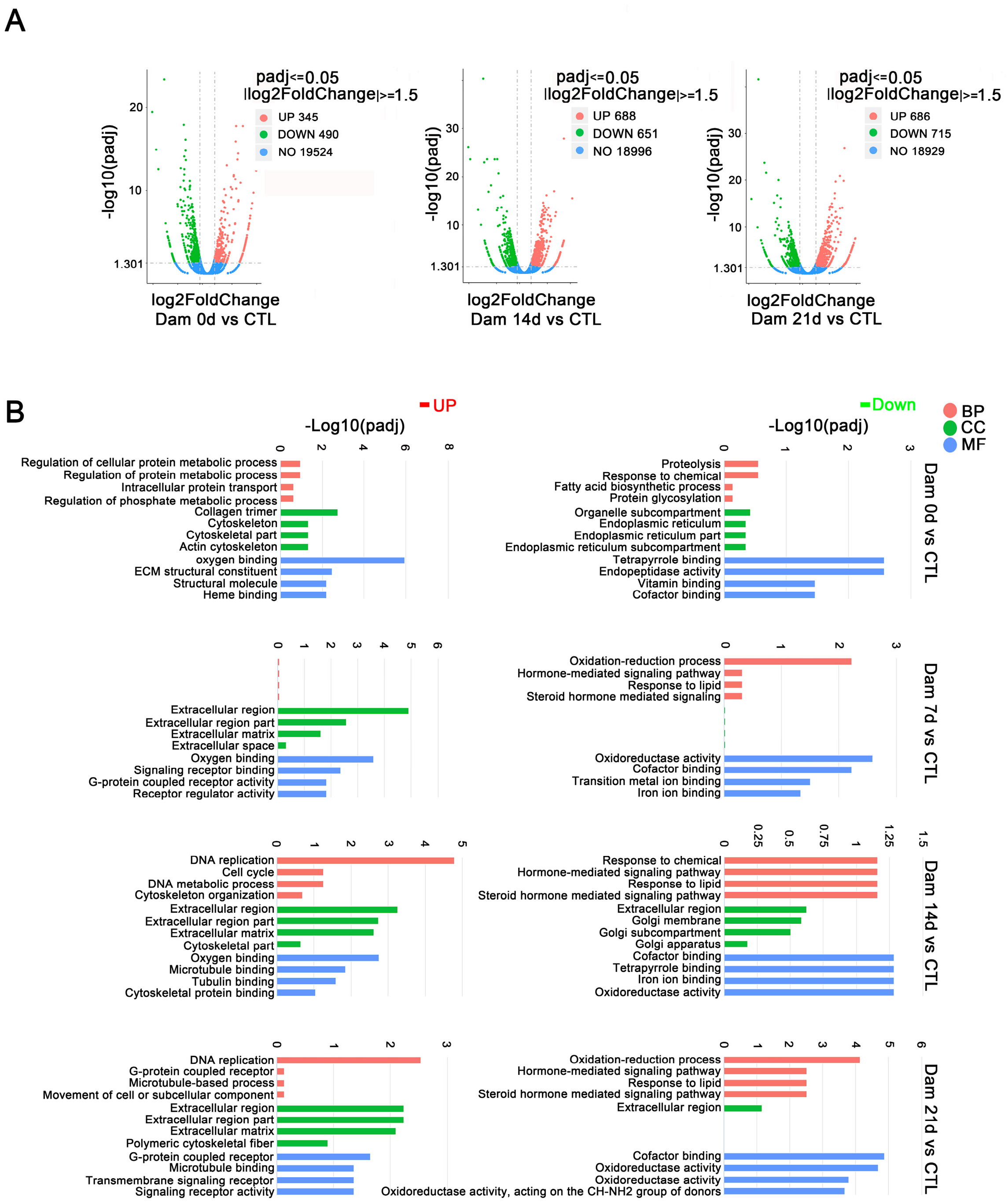

Supplementary Figure S3. RNA-seq results of skin tissue after epidermal damage in lamprey. (A) Volcano plot of the differentially expressed genes in lamprey after skin damage at all five time points. (B) GO functional category analysis of all five time points (from 0 d-21 d vs. CTL), showing upregulated DEGs (left) and downregulated DEGs (right).

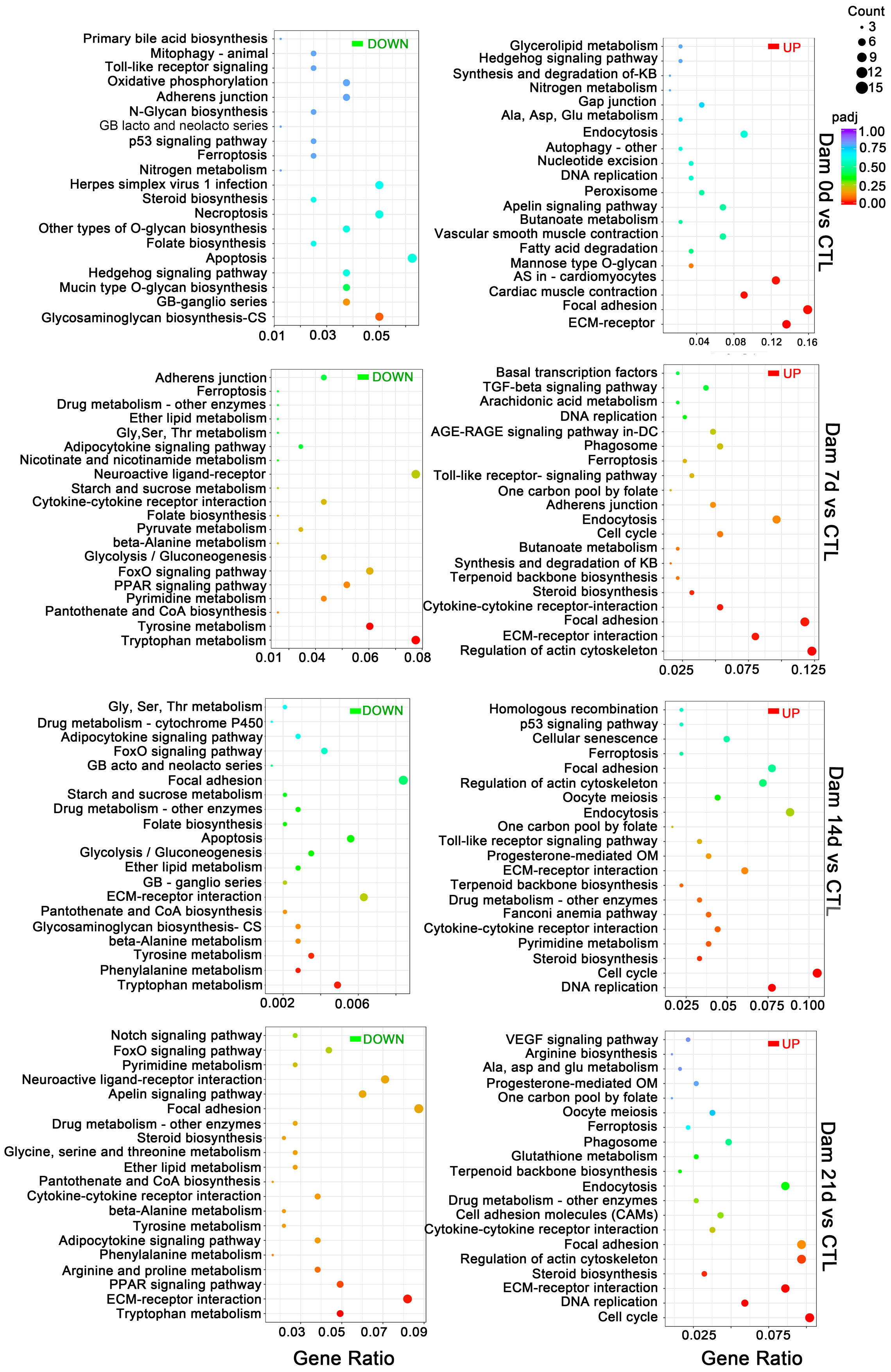

Count
3
6
9
12
15
padj
1.00
0.75
0.50
0.25
0.00
Primary bile acid biosynthesis
Mitophagy - animal
Toll-like receptor signaling
Oxidative phosphorylation
Adherens junction
N-Glycan biosynthesis
GB lacto and neolacto series
p53 signaling pathway
Ferroptosis
Nitrogen metabolism
Herpes simplex virus 1 infection
Steroid biosynthesis
Necroptosis
Other types of O-glycan biosynthesis
Folate biosynthesis
Apoptosis
Hedgehog signaling pathway
Mucin type O-glycan biosynthesis
GB-ganglio series
Glycosaminoglycan biosynthesis-CS
DOWN
0.01
0.03
0.05
Glycerolipid metabolism
Hedgehog signaling pathway
Synthesis and degradation of-KB
Nitrogen metabolism
Gap junction
Ala, Asp, Glu metabolism
Endocytosis
Autophagy - other
Nucleotide excision
DNA replication
Peroxisome
Apelin signaling pathway
Butanoate metabolism
Vascular smooth muscle contraction
Fatty acid degradation
Mannose type O-glycan
AS in - cardiomyocytes
Cardiac muscle contraction
Focal adhesion
ECM-receptor
UP
0.04
0.08
0.12
0.16
Dam 0d vs CTL
Adherens junction
Ferroptosis
Drug metabolism - other enzymes
Ether lipid metabolism
Gly,Ser, Thr metabolism
Adipocytokine signaling pathway
Nicotinate and nicotinamide metabolism
Neuroactive ligand-receptor
Starch and sucrose metabolism
Cytokine-cytokine receptor interaction
Folate biosynthesis
Pyruvate metabolism
beta-Alanine metabolism
Glycolysis / Gluconeogenesis
FoxO signaling pathway
PPAR signaling pathway
Pyrimidine metabolism
Pantothenate and CoA biosynthesis
Tyrosine metabolism
Tryptophan metabolism
DOWN
0.01
0.04
0.06
0.08
Basal transcription factors
TGF-beta signaling pathway
Arachidonic acid metabolism
DNA replication
AGE-RAGE signaling pathway in-DC
Phagosome
Ferroptosis
Toll-like receptor- signaling pathway
One carbon pool by folate
Adherens junction
Endocytosis
Cell cycle
Butanoate metabolism
Synthesis and degradation of KB
Terpenoid backbone biosynthesis
Steroid biosynthesis
Cytokine-cytokine receptor-interaction
Focal adhesion
ECM-receptor interaction
Regulation of actin cytoskeleton
UP
0.025
0.075
0.125
Dam 7d vs CTL
Gly, Ser, Thr metabolism
Drug metabolism - cytochrome P450
Adipocytokine signaling pathway
FoxO signaling pathway
GB acto and neolacto series
Focal adhesion
Starch and sucrose metabolism
Drug metabolism - other enzymes
Folate biosynthesis
Apoptosis
Glycolysis / Gluconeogenesis
Ether lipid metabolism
GB - ganglio series
ECM-receptor interaction
Pantothenate and CoA biosynthesis
Glycosaminoglycan biosynthesis- CS
beta-Alanine metabolism
Tyrosine metabolism
Phenylalanine metabolism
Tryptophan metabolism
DOWN
0.002
0.006
Homologous recombination
p53 signaling pathway
Cellular senescence
Ferroptosis
Focal adhesion
Regulation of actin cytoskeleton
Oocyte meiosis
Endocytosis
One carbon pool by folate
Toll-like receptor signaling pathway
Progesterone-mediated OM
ECM-receptor interaction
Terpenoid backbone biosynthesis
Drug metabolism - other enzymes
Fanconi anemia pathway
Cytokine-cytokine receptor interaction
Pyrimidine metabolism
Steroid biosynthesis
Cell cycle
DNA replication
UP
0.025
0.05
0.075
0.100
Dam 14d vs CTL
Notch signaling pathway
FoxO signaling pathway
Pyrimidine metabolism
Neuroactive ligand-receptor interaction
Apelin signaling pathway
Focal adhesion
Drug metabolism - other enzymes
Steroid biosynthesis
Glycine, serine and threonine metabolism
Ether lipid metabolism
Pantothenate and CoA biosynthesis
Cytokine-cytokine receptor interaction
beta-Alanine metabolism
Tyrosine metabolism
Adipocytokine signaling pathway
Phenylalanine metabolism
Arginine and proline metabolism
PPAR signaling pathway
ECM-receptor interaction
Tryptophan metabolism
DOWN
0.03
0.05
0.07
0.09
Gene Ratio
VEGF signaling pathway
Arginine biosynthesis
Ala, asp and glu metabolism
Progesterone-mediated OM
One carbon pool by folate
Oocyte meiosis
Ferroptosis
Phagosome
Glutathione metabolism
Terpenoid backbone biosynthesis
Endocytosis
Drug metabolism - other enzymes
Cell adhesion molecules (CAMs)
Cytokine-cytokine receptor interaction
Focal adhesion
Regulation of actin cytoskeleton
Steroid biosynthesis
ECM-receptor interaction
DNA replication
Cell cycle
UP
0.025
0.075
Gene Ratio
Dam 21d vs CTL

Supplementary Figure S4. RNA-seq results of skin tissue after epidermal damaged in lamprey. KEGG analysis of all five time points (from Dam 0 d-21 d vs. CTL). The left side shows the upregulated DEGs, and the right side shows the downregulated DEGs. GB: Glycosphingolipid biosynthesis; CS: chondroitin sulfate.

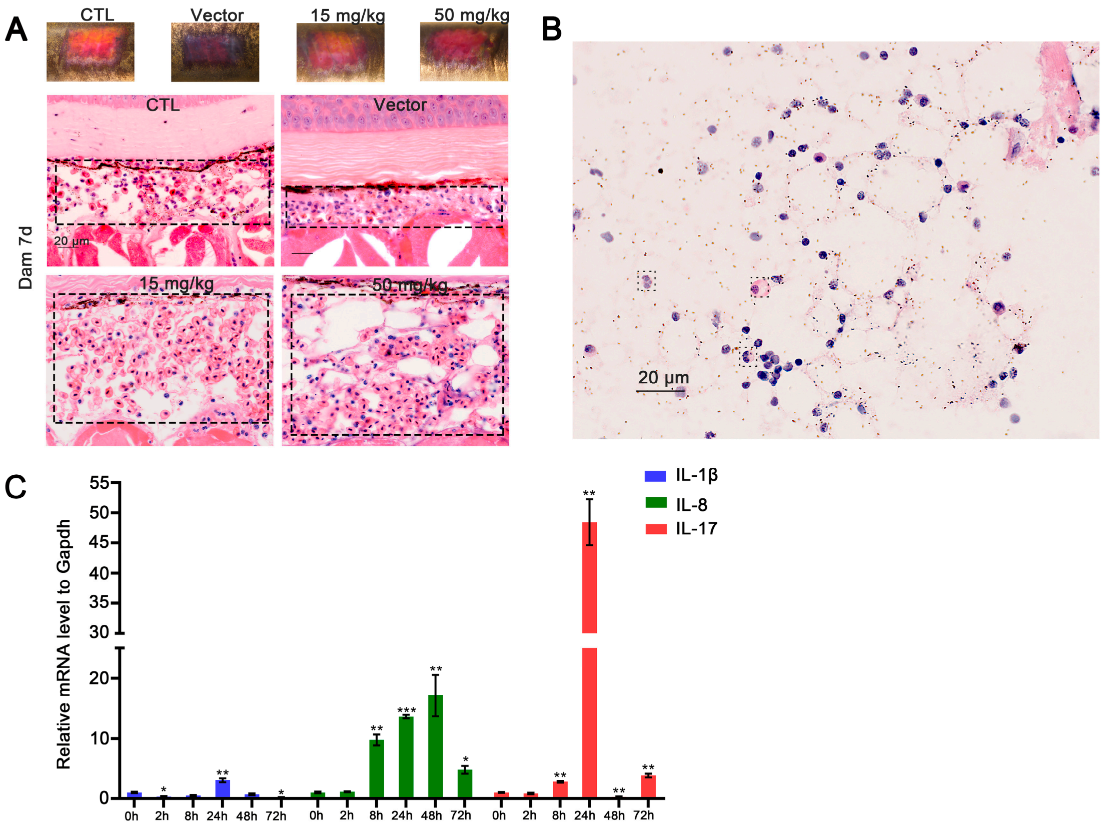

Supplementary Figure S5. (A) Effect of RAC1 inhibitor addition on epidermal repair. Dam 7 d after addition of the Ehop-016 inhibitor. Early hypodermis changes were relieved on Dam 7 d (black box). Scale bars, 20 μm. (B) Hypdodermis smear of epidermal damage and different types of cell infiltration (black box). (C) Isolated RBCs stimulated by Vibrio anguillarum can promote inflammation. * $p < 0.05$, ** $p < 0.01$, *** $p < 0.001$.
