# Supplementary material for: Lamprey Wound Healing and Regenerative Effects: The Collaborative Efforts of Diverse Drivers
Source: Int J Mol Sci. 2023 Feb 6;24(4):3213. doi: 10.3390/ijms24043213 (PMC9965152; doi:10.3390/ijms24043213)
Supplement: Supplementary file 1 [file ijms-24-03213-s001.zip › Supplementary Table.pdf]

Supplementary Table S1 qPCR primer sequence

Gene name	F-terminal primer	R-terminal primer
ACTB2-like	GGCACAGAGCAAGCGTGGTATC	GATCTTCTCCATGTCGTCCCAGTTG
FAK1-like	TCATGGTGGTGAGCAGAGACTCC	CAACATCCAGCGGACCTACAACAG
VAVA3	GCAGGATGACTCGCAGGTGTTC	CTCAAGCACAGGAACTGGGACATC
DOCK1-like	TGGAGGAGCAGATACAGGAGAAGTC	GTACGAGTGAGTGGCGGAGAAATG
RHOA-like	GCTTGCCGTCCACCTCAATGTC	ATCGTGTTCAGCAAGGACCAGTTC
FGF9-like	GCGTAGTGAAGTACCGTCGAGTAAC	GCACACACATCCCTTTTGTCAATCC
FACK-1-like	TCATGGTGGTGAGCAGAGACTCC	CAACATCCAGCGGACCTACAACAG
PDGFRA-like	CAGCGTCGGTCATACAGGAGTTG	TTGCTCTTCTCGTCGTTGCCATC
ITGA11-like	TCACGCAGATGGAGTCCTTCCC	CATGAACGCCACGGTGACCTTC
RRAS2	CGGATTTGGAGGAAAGCGGATACC	TGGAAAGGGCAAGAGGAGGAAGG
ITGA8-like	TCTGGATGTGGTCTGCCTGGTAG	GTGTCTCTGAACTACAGCCTGGATG
COL2A1	TCATCGCCGTAGTTGAAGTGTAAGC	CACAGACCATCTCCCGCAAGAAC
Fgf4a-like	ACTGTATCGACTCGGCCGTG	CCCTTGCTGAGGCCGATGTA
RAC1	AAGCGATTAAGTGCGTGGTGGTC	GGGAAGGCGTTTGTGGTGTAGC
ARP2/3	CATGATGATGCGGGCGGAGAAC	CGTGTGGAAGTTGGTGATGAGGAAG

Supplementary Table S2 qPCR primer sequence

Gene name	F-terminal primer	R-terminal primer
BAL	GCCAGCTTACTCGACAACTTCCTC	ACGGTAGTTGAAGGTCACCACAATG
ATGL	CTTCTGCTGGTTCCGTGCTCTG	GCTCTTCCTCCTCCTCCTCCTC
PNLIPRP2-like	CCTACTCGGGCTACACAGAGAGG	GGGTTGGCTTTGGTGAAGAGGAG
DGL	CAAGACTGCTACGCTCACAAGGG	TGGTAATCCTGTGCCTGCTGAAAC
MGL	CCGACAAGACGCTGCACATCTAC	GCCACGATCCAAGCCTCAATCTC
Il-17	TGCTGGCGGTGTCTGCTTGGCTCGT	CTCTAGGTCCGGCTTGTAAACGTA
IL1-1-like	CCAGGAGGGCAACGCACATC	CGGAACAGGTCGCTGCTCTTG
IL-8	AAGCTCCTTGTCGTCCTTCTCGCC	TTACTGCGTGGATTTGGGTGTCTG
